# Zeaxanthin‐Producing *Winogradskyella schleiferi* Strains

**DOI:** 10.1002/jobm.70187

**Published:** 2026-07-17

**Authors:** Aldo Betancourt Sanchez, Alexander Huynh, Laurence Blanchard, Arjan De Groot, Christophe Klopp, Karine Loubière, Caroline Andriantsiferana, Barbora Lajoie

**Affiliations:** ^1^ Laboratoire de Génie Chimique (LGC) Université de Toulouse, Toulouse INP, CNRS Toulouse France; ^2^ CEA, CNRS, BIAM, Molecular and Environmental Microbiology (MEM) Team, Aix Marseille Université Saint Paul‐Lez‐Durance France; ^3^ INRAE, Genotoul Bioinformatics Platform, Applied Mathematics and Informatics of Toulouse, Sigenae, MIAT, UR875 Castanet Tolosan France

**Keywords:** carotenoids, marine bacteria, zeaxanthin

## Abstract

Two pigmented bacteria strains were isolated from the Mediterranean Sea. Phylogenetic analysis revealed that both strains are affiliated with *Winograsdskyella schleiferi* Z215. However, in contrast to this reference strain, they can assimilate additional carbon sources such as d‐mannose, l‐arabinose, d‐mannitol, and citrate. Zeaxanthin was identified as the major carotenoid produced by both strains, predominantly in the *all‐trans* configuration. Carotenoid biosynthesis kinetics revealed that pigment production commenced at the early stages of growth, with maximal yields reached between 48 and 56 h (between 1 and 1.3 mg L^−1^). Specific production yields were 0.7 and 0.9 mg g^−1^ for the two strains, respectively. Total carotenoid production was influenced by incubation temperature in both strains. Overall, these findings expand the scientific knowledge on carotenoid‐producing marine bacteria and highlight the potential of these newly isolated strains as promising candidates for zeaxanthin biotechnological production for a wide range of novel applications ranging from feed additives to treatments for macular degeneration and melanoma.

**Abbreviations**
CFU: colony‐forming units
DCM: dichloromethane
EPS: extracellular polymeric substances
Fr: frontal ratio
HPLC: high‐pressure liquid chromatography
LC‐MS: liquid chromatography mass spectrometry
OD: optical density
rRNA: ribosomal ribonucleic acid
Rt: retention time
TLC: thin‐layer chromatography
*µ*_max_
: maximum specific growth rate

## 1 Introduction

Carotenoids are natural pigments belonging to the terpenoid family, derived from isoprenoids. Algae, plants, fungi, and bacteria are among the living organisms capable of synthesizing these molecules, which display colors ranging from yellow to red. These compounds play key biological roles, including transferring light energy to chlorophyll and protecting cells against photo‐oxidative damage by neutralizing free radicals [1]. They are classified into two main groups: carotenes (purely hydrocarbon chains containing 30 to 60 carbon atoms) and xanthophylls (containing oxygenated functional groups: hydroxy, epoxy, keto, methoxy, or carboxylic groups) [2].

The main carotenoids produced industrially are lycopene, β‐carotene, astaxanthin, lutein and canthaxanthin (Figure S1). Their annual worldwide consumption was estimated to be around 5693 and 6222 metric tons in 2017 and 2022, respectively [3].

Among carotenoids, zeaxanthin (3R,3′R‐*β*,*β*‐carotene‐3,3′‐diol) is a niche but particularly valuable carotenoid. It is a yellow xanthophyll with the molecular formula C_40_H_56_O_2_ and a molecular weight of 568.88 g mol^−1^. It is naturally found in marigold flowers, corn, egg yolks, and alfalfa and it is also produced by various microorganisms such as microalgae, filamentous fungi and bacteria [4]. Zeaxanthin is industrially used as a colorant in poultry and fish food to enhance skin pigmentation [4, 5]. In medicine, together with lutein, it plays a critical role in preventing age‐related macular degeneration, a leading cause of blindness [6], and has also been investigated for its potential to induce apoptosis in melanoma cells [7].

Currently the main zeaxanthin production method is plant extraction from yellow vegetables and fruits [8]. However, this method is high‐cost, energy‐consuming, and low‐yield due to the low zeaxanthin content in plant biomass (around 0.3 mg g^−1^ of marigold flower) [9, 10]. One alternative is chemical synthesis by a Wittig condensation, with C‐10 aldehyde as the central building block and two equivalents of C‐15 phosphonium salt, which allows high control over purity [10]. However, this approach generates undesirable S‐S and S‐R stereoisomers and unoxidized forms. The other alternative is bioproduction using bacteria, filamentous fungi, yeast and microalgae, which produce the principal natural (3R,3′R)‐zeaxanthin isomer and offer easier processing than plants [11]. Both microalgae and bacteria are the most common naturally zeaxanthin‐accumulating microorganisms [9]. Microalgae such as *Chlorella, Spirulina, Dunaliella* can accumulate up to 11.2 mg g^−1^ dry biomass [12], but production costs are high due to nutrient‐rich growth media and energy costs associated with light exposure for photosynthesis. Similar limitations apply to some bacteria [13].

In this context, marine bacteria are a promising alternative to microalgae for carotenoid production, as they can synthesize bioactive carotenoids while being able to survive in low nutrient conditions which are hostile to microalgae [14]. Moreover, their tolerance to high‐salinity reduces contamination risk, making them advantageous for large‐scale carotenoid production [15]. *Winogradskyella* is a genus of pigmented marine bacteria whose potential for carotenoid synthesis has yet to be thoroughly studied. In this regard, this study aims to characterize 2 novel *Winogradskyella* strains isolated from the Mediterranean Sea and evaluate their potential for zeaxanthin production.

## 2 Materials and Methods

### 2.1 Bacterial Strain Isolation and Characterization

Microbial biofilm samples were taken from seawater in Genoa harbor, Italy [16]. From these samples, isolates forming yellow colonies on marine agar plates were selected, as they were expected to be potential candidates for zeaxanthin production. The two selected strains were referred to as strains A and B.

Phenotypic characterization was performed using the API 20NE gallery (bioMérieux SA). Each strain was prepared in synthetic marine medium to a turbidity equal to 0.5 McFarland standard and inoculated according to the manufacturer's instructions. Enzymatic activities were analyzed after 24 h, and carbon source utilization was read after 3 days of incubation. Catalase activity was determined by measuring the production of oxygen bubbles in 3% (v/v) hydrogen peroxide and oxidase activity by color change after adding a droplet of *N*,*N*‐dimethylphenylenediamine. Gram staining was also performed on both strains. Growth of both strains at different temperatures (4°C, 20°C, 30°C, and 37°C) was evaluated using 24‐well microplates.

Antibiotic susceptibility was tested in triplicate after spreading cells (100 µL with an optical density (OD) at 640 nm of 0.1) on marine agar plates followed by adding disks of gentamycin (10 µg), penicillin (6 µg), ampicillin (2 µg) and erythromycin (15 µg) on the surface and incubation for 4 days at 20°C.

Cell morphology was analyzed by scanning electron microscopy at the Center for Electron Microscopy Applied to Biology (Toulouse, France). Bacterial autofluorescence was observed with an epifluorescence microscope Carl ZEISS Axio Imager‐M2. Filter SYTO9 and PI were used to observe green and red fluorescence respectively. The objective used was ZEISS EC Plan‐neofluar 20× and pictures were taken with a ZEISS Axiocam Cm1 camera.

### 2.2 DNA Isolation and 16S rRNA Gene Analysis

Genomic DNA was extracted from isolated colonies grown on marine broth agar using the Promega genomic DNA kit (Omega Bio‐Tek, USA). 16S rRNA gene sequencing was performed at the Genotoul platform (INRA Castanet, France). Sequencing quality and depth were checked with GenomeScope2 [17]. Reads were assembled using SPAdes (v.3.15.5) [18] with default settings. The resulting contigs were compared to the complete genome of *W. schleiferi* strain Z215 (NZ_CP053351.1) using D‐GENIES [19]. The rRNA gene was searched with minimap2 [20] by aligning to the public *W. schleiferi* strain Z215 rRNA reference NR_179473.1 and extracted using the alignment coordinates with samtools faidx [21] version 1.19 with default parameters.

A phylogenetic tree was constructed using the MEGA 12.0 program and the Maximum Likelihood method [22]. The 16S rRNA gene sequences of strains A and B were deposited in GenBank database under accession numbers PZ043966 and PZ051820, respectively. Accession numbers for the 16S rRNA sequences from the other bacteria in the phylogenetic tree, chosen due to their highest similarity to the 16s rRNA of strains A and B, were recovered from a prokaryotic strain database “BacDive” [23], which are LR745724 (*W. schleiferi* Z215), MW422811 (*W. marina*), LR745719 (*W. ludwigii*), LR745723 (*W. forsetii*) FJ595484 (*W. exilis*), GQ181061 (*W. pacifica*), LR745714 (*W. wichelsiae*), LR745715 (*W. costae*), and LR745718 (*W. vidalii*).

### 2.3 Cultivation Conditions

All bacterial manipulations were performed under sterile conditions. Cryopreserved *W. schleiferi* strains were first activated in 10 mL of commercial marine broth (Table S1) on a rotatory shaker (200 rpm) for 2 days at 30°C (strain A) and at 20°C (strain B). The preculture was incubated in 100 mL Erlenmeyer flasks for 2 days at 100 rpm. A final 1 L culture was prepared in 2 L Erlenmeyer flasks with an initial OD adjusted to 0.1 to facilitate adaptation before exponential growth [24]. Cultures were incubated for 56 h. Bacterial growth was monitored by three methods: OD, dry biomass and colony‐forming units (CFU mL^−1^). OD was measured in a spectrophotometer by taking samples at regular intervals. To estimate the number of CFU mL^−1^, 1 mL samples were taken at 0, 8, 24 and 48 h. Successive 1/10th dilutions were prepared in a phosphate‐buffered saline solution. The appropriate dilutions were each spread on marine broth agar plates, which were then incubated for 4 days at 20°C. All experiments were done in duplicate.

Microbial contamination was checked daily by plating culture droplets on Trypticase‐Soy Agar and incubating at 37°C for 24 h. Colony growth indicated contamination, as *W. schleiferi* does not grow without mineral salts.

### 2.4 Carotenoid Extraction

After cultivation, the bacterial suspension was centrifuged at 3270 g for 30 min at 4°C. The supernatant was discarded and the cell pellet was washed twice with distilled water to remove residual salts. Carotenoids were extracted by resuspending the cell pellet in 15 mL of 96% ethanol followed by incubation for 15 min at 20°C in an ultrasonic bath with intermittent mixing. After centrifugation, the supernatant was collected and the absorbance spectrum was recorded between 350 to 600 nm. These steps were repeated until the cells were completely bleached. The remaining cell debris was used for biomass measurement (see Section 2.5).

The carotenoid mass expressed in terms of *zeaxanthin equivalent*, *M*_c_, was estimated by using Equation (1), which derives from the Beer‐Lambert equation:

(1) $$M_{C}\;=\;\frac{\text{Abs}\;\times \;V_{\text{extract}}\times M}{\varepsilon \times l\;}$$

where Abs is the absorbance measured at 450 nm of the ethanolic solution containing the carotenoids after ultrasonication, *V*_extract_ the sample volume (mL), *M* the molar mass of zeaxanthin (566.8 g mol^−1^), *l* the optical pathlength of the cuvette used (1 cm) and *ε* the zeaxanthin specific molar absorption coefficient in ethanol at 450 nm equal to 1.45 × 10^5^ (L mol^−1^ cm^−1^) [25].

Afterwards, ethanol was evaporated under reduced pressure at 30°C. Major carotenoids were isolated from the dried extract by dissolving it in dichloromethane (DCM) and performing thin‐layer chromatography (TLC) on a silica plate using a mixture of DCM and ethyl acetate (4:1 v/v). The yellow band appearing at a frontal ratio (Fr) of 0.42 was isolated and the silica matrix was washed with acetone to recover the target carotenoid. The acetone was then evaporated and the pigment was resuspended in methanol for liquid chromatography‐mass spectrometry (LC‐MS) analysis.

For high‐performance liquid chromatography (HPLC) analysis, dried extract obtained after ethanol evaporation was resuspended in methanol and filtered through a 0.2 µm membrane.

### 2.5 Analytical Tools

Dry biomass was measured after drying the cell debris in a convection oven at 105°C for 24 h. Pigment absorbance was measured using a Helios beta UV–Vis spectrophotometer. HPLC analyses were carried out in a Vanquish system equipped with a Luna Omega C18 column (3 µm, 150 × 2.1 mm), using a UV–Vis photodiode array detector. Methanol was used as the mobile phase (0.3 mL min^−1^, 10 µL injection). Chromatograms were recorded at 450 nm. Carotenoid standards were purchased from Sigma‐Aldrich. LC‐MS analyses were conducted at the technical and scientific platform of the Institut de Chimie de Toulouse (ICT, France) using a DSQ II simple quadrupole with direct chemical ionization.

## 3 Results

### 3.1 Isolation and Identification of *W. schleiferi* Strains

**Figure 1:**
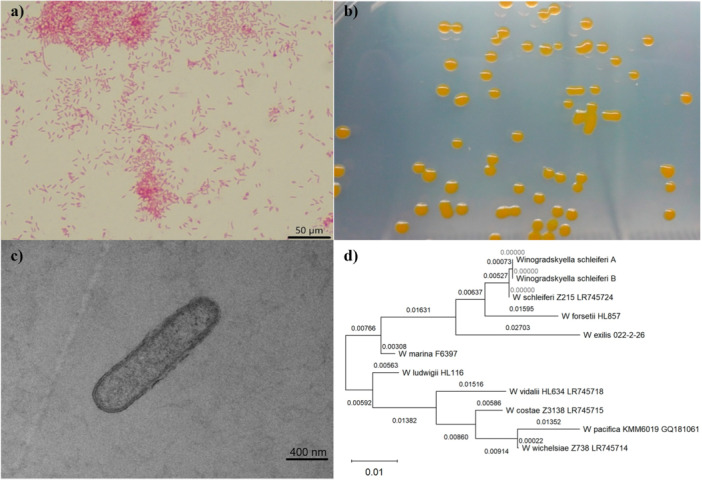
Cells, colonies and phylogenetic analysis of novel *Winogradskyella schleiferi* strains. (a) Gram stain of *W. schleiferi* A, (b) marine broth culture plate showing colonies of *W. schleiferi* strain A, (c) scanning electron micrograph of *W. schleiferi* A the same results were observed for strain B, (d) phylogenetic tree of 16S rRNA gene sequence of *W. schleiferi* strains A and B, and related taxa.

The two microbial isolates recovered from seawater biofilm are Gram negative cells (Figure 1a), forming round orange–yellow colonies (Figure 1b). They are mainly rod‐shaped with some coccus forms spotted occasionally (Figures 1c and S2) and are strictly aerobic. Additionally, they are mesophilic having an optimal growth between 20°C and 30°C and grow in media containing 19 g L^−1^ of NaCl. Epifluorescence microscopy allowed for observation of pigments contained inside the bacteria (Figure S3).

Sequence comparisons (using minimap2) and phylogenetic analysis revealed that the 16S rRNA gene sequences of strains A and B are identical, and have the highest similarity (99.93%) with *Winogradskyella schleiferi* strain Z215 (Figures 1d and S4). This work, to be best of our knowledge, is the first to report the presence of *W. schleiferi* in the Mediterranean Sea, whereas previous publications on this species reported its presence only at the Helgoland site in the North Sea [15].

The phenotypic characteristics of the strains A and B and the comparison with other *Winogradskyella* species reported in the literature are shown in Table 1 [26, 27, 28]. The choice of the references was made due to their proximity to *W. schleiferi* on the phylogenetic tree.

**Table 1:** Comparison of the phenotypic characteristics of the strains A and B with other *Winogradskyella* species, i.e., *W. schleiferi, W. forsetii, W. ludwigii* [26], *W. exilis* [27], *W. marina* [28].

	Strain A	Strain B	*W. schleiferi* Z215	*W. forsetii* HL857	*W. exilis* 022‐2‐26	*W. marina* F6397	*W. ludwigii* H116
Activity:							
Catalase	+	+	+	+	+	+	+
Oxidase	−	−	−	+	−	+	−
Nitrate reductase	−	−	−	−	−	n.d.	+
β‐Galactosidase	−	−	−	+	−	n.d.	−
β‐Glucosidase	+	+	−	−	n.d.	n.d.	−
Utilization of:							
d‐glucose	−	−	−	−	−	+	−
d‐mannose	−	+	−	−	−	+	−
d‐mannitol	+	+	−	−	−	+	−
l‐arabinose	−	+	−	−	n.d.	n.d.	−
Citrate	+	+	−	−	n.d.	−	−
Tween 20	+	+	+	+	n.d.	+	+
Susceptibility to:							
Ampicillin	−	−	n.d.	n.d.	+	+	n.d.
Gentamycin	−	−	n.d.	n.d.	−	−	n.d.
Penicillin	−	−	n.d.	n.d.	n.d.	+	n.d.
Erythromycin	+	+	n.d.	n.d.	−	+	n.d.

Strains A and B possess catalase and oxidase activity, just like most of the other species, as well as β‐glucosidase activity, unlike *W. schleiferi* Z215. Concerning the utilization of carbon sources, differences were observed with strain Z215, but also between the new strains A and B. Acidification of the medium was observed with citrate and mannose but not with mannitol nor arabinose (Table S2). Strains A and B were resistant gentamycin, penicillin and ampicillin, but were susceptible to erythromycin.

Both strains grew optimally at 20°C to 30°C, but the highest pigment production was recorded at 30°C and at 20°C for strains A and B respectively (Figure S5).

### 3.2 Identification of Carotenoids Extracted from *W. Schleiferi* A and B

**Figure 2:**
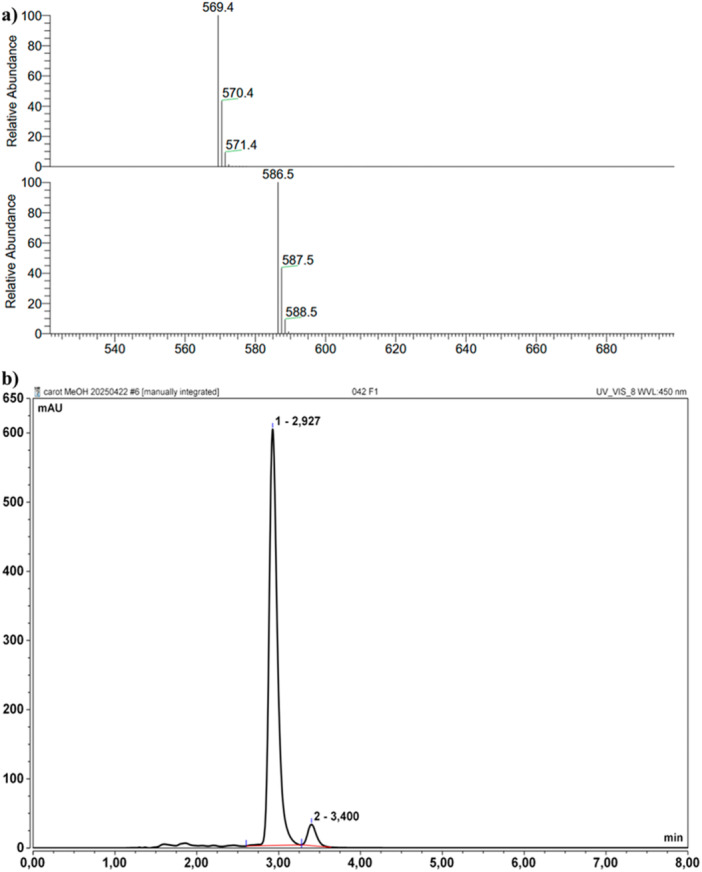
(a) MS analysis and (b) HPLC elution profile of isolated pigment (Fr 0.42) dissolved in methanol for strain B. The same results were observed for strain A.

The separation of the different carotenoids contained in the total extract of both strains was performed using TLC (Figure S6a). The spectrophotometric analysis showed that the isolated pigment had a maximum absorption peak at 450 nm (II) with two shoulder peaks at 420 nm (I) and 475 nm (III) (Figure S6b). The mass spectrometry analysis (Figure 2a) showed the protonated molecule [M + H]^+^ at m/z 569.5 and the ion [M + NH_4_]^+^ at m/z 586.5, both corresponding to zeaxanthin's molecular weight. The HPLC chromatogram (Figure 2b) revealed two peaks appearing at retention times (Rt) equal to 2.9 and 3.4 min, where absorbance spectra correlate to *all‐trans* and *cis* isomers of zeaxanthin, respectively [5]. The isomers can be distinguished by the additional peak that appeared at 274 nm for the *trans* isomer and at 336 nm for the *cis* isomer (Figure S7). The ratio of the height of the longest wavelength absorption peak (III) to that of the middle absorption peak (II), %III/II, was 23% and 15% for the *trans* and *cis* isomer respectively, which correspond to previously reported values [5, 26]. These findings confirm the ability of *W. schleiferi* A and B to produce zeaxanthin.

**Figure 3:**
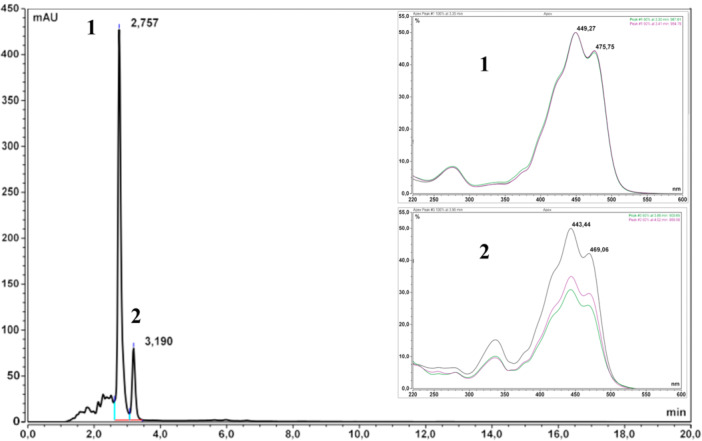
Example of HPLC elution profiles (450 nm) of the total carotenoid extract produced by strain B. The absorption spectra profiles of the main peaks identified by HPLC are also inserted.

As shown in Figure 3, two main pigments were identified on the total carotenoid extract of strain B at a Rt of 2.7 and 3.2 min. Their absorption spectra profiles revealed two merged peaks with a side shoulder, which correspond to *all‐trans* and *cis* zeaxanthin isomers, respectively [26], the former showing the highest intensity. The same main carotenoids were identified on strain A (Figure S8), indicating that zeaxanthin is the main carotenoid synthesized by these strains of *W. schleiferi*.

### 3.3 Carotenoid Production by *W. Schleiferi* A and B

**Figure 4:**
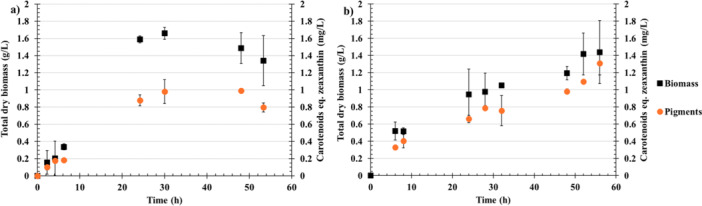
Biomass growth kinetics expressed in dry biomass and zeaxanthin equivalent production kinetics for (a) strain A and (b) strain B. The error bars represent the standard deviation between the tests' values (*n* = 2).

In Figure 4, the kinetics of both microbial growth and carotenoid production (expressed as equivalent zeaxanthin) are reported for strain A and strain B. Strain A had an adaptation phase lasting almost 6 h, followed by the exponential phase, during which peak biomass concentration was achieved after the first day of incubation. After this, the decline phase was observed at 52 h. The generation time was 3.8 h and the maximum specific growth rate (*µ*_max_) was 0.18 h^−1^. A maximum dry biomass of 1.7 ± 0.1 g L^−1^ was obtained after 30 h. For strain B, the adaptation and exponential phase durations were similar to those of strain A; however, the decline phase was not observed during the experiment (56 h). Additionally, strain B had a generation time of 6.4 h and a *µ*_max_ of 0.11 h^−1^. The maximum dry biomass was obtained at 56 h, yielding 1.4 ± 0.4 g L^−1^.

Concerning carotenoid production, a significant increase was observed for strain A during the first 24 h, achieving a concentration of 0.88 ± 0.06 mg L^−1^. This production slowed down until reaching a maximum concentration of 0.99 ± 0.01 mg L^−1^ after 48 h of culture. For strain B, carotenoid production increased almost linearly with time, leading to 1.31 ± 0.14 mg L^−1^ after 56 h. The specific production for strain B, expressed in mg carotenoids eq. zeaxanthin per g dry biomass, was 0.9 mg g^−1^ while for strain A it was 0.7 mg g^−1^.

## 4 Discussion

The novel Mediterranean strains of *W. schleiferi* are remarkable for their ability to consume a wider variety of carbon sources compared to the previously reported strain Z215 from the North Sea. This versality can improve the cultivation conditions of these bacteria making them interesting candidates for sustainable biotechnological carotenoid production. Additionally, these bacteria have been shown to produce mainly zeaxanthin which, to the best of our knowledge, has not yet been studied profoundly in the literature for *W. schleiferi*. Previous publications where the *Winogradskyella* genus has appeared have not delved deeper into the identification of the main carotenoids nor into the production kinetics of these compounds, hence the interest of this research. Several aspects remain to be explored in the future to evaluate the feasibility of large‐scale zeaxanthin production with these strains, notably: metabolic pathways, biomass recovery, and yield optimization.

Zeaxanthin is synthesized from β‐carotene by β‐carotene hydroxylase which is encoded by the gene *crtZ* in bacteria [29]. This gene was identified in *W. schleiferi* A and B using the gene prediction tool Prokka. Additionally, to delve further into the regulation mechanisms, the proteorhodopsin gene “*prd*,” which codes for light‐energy‐harvesting transmembrane proteins, was also identified in both strains. A study tied the presence of this gene and the gene cluster for zeaxanthin synthesis in 37 strains of flavobacterium where a transcriptomic analysis determined that light induced expressions of both the zeaxanthin and proteorhodopsin genes [30]. To further describe this metabolic relationship, a deeper study focusing on the effect of light on zeaxanthin production in *W. schleiferi* has to be made separately.

Another metabolic phenomenon of interest is the role of zeaxanthin in cell development. Interestingly, Figure 4 shows that the kinetics of the carotenoid production followed a similar trend to the biomass growth kinetics. This correlation suggests that zeaxanthin could be a primary metabolite, but more experiments focusing on different culture conditions should be done to confirm this hypothesis. From a large‐scale production perspective, a primary carotenoid could be a key advantage because, in theory, maximizing biomass growth would also maximize carotenoid production. Such a pattern contrasts with typical secondary metabolites of marine bacteria (e.g., antimicrobial compounds like bacitracin A and phenylacetic acid [31], which are mainly synthesized under stress conditions that can lead to death). When targeting a primary carotenoid, the maximum biomass concentration serves as an indicator to proceed to biomass recovery and carotenoid extraction.

During biomass recovery, strain A was markedly easier to process than strain B. While one centrifugation was enough to recover all biomass from the strain A culture, this was not enough for strain B as the supernatant still retained an intense orange coloration. A possible explanation is the production of extracellular polymeric substances (EPS) which can form a network between the cells, keeping them in suspension, as observed for *Winogradskyella thalassocola* [32]. For this reason, the EPS were precipitated by adding 96% ethanol to the supernatant in a 1:1 (v/v) ratio for strain B, such as described in [33]. Once this EPS precipitate was recovered, the same protocol for carotenoid extraction (Section 2.4) was applied. Therefore, for strain B, the total dry biomass shown in Figure 4 corresponds to the sum of the cell debris and EPS precipitate. In large scale production, EPS should be taken into account and ideally restricted to avoid the introduction of a precipitation step to the downstream process. Alternatively, the EPS can be valorized (after pigment extraction) for other purposes like biomineralization [34].

To date, there are no studies that report the carotenoid‐producing potential of the *Winogradskyella* genus. The strains studied in this work were able to produce high quantities of zeaxanthin after 2 days of culture: for strain A, 0.7 mg g^−1^ dry biomass after 48 h and for strain B, 0.9 mg g^−1^ dry biomass after 56 h. These results can be compared with the wide range of yields of other marine microorganisms.

Bacteria from the *Flavobacterium* genus produce zeaxanthin with high yields; for instance, *Flavobacterium* sp. produces up to 16 mg g^−1^ after 48 h [35], while *Flavobacterium multivorum* produces only 1.6 mg g^−1^ of zeaxanthin [36]. The production by *W. schleiferi* falls in the same range as the one of bacteria like *Cyanobium* sp., *Nubsella zeaxanthinifaciens*, *Mesoflavibacter zeaxanthinifaciens* which have yields between 0.8 and 1.06 mg g^−1^ [37, 38]. The use of different substrates and stress induction through different abiotic parameters, such as light irradiation or temperature, could improve *W. schleiferi* carotenoid production [39].

Microalgae like *Synechoccus* sp. and *Chlorella* sp. have shown yields between 3 and 11 mg g^−1^ [13]. In photosynthetic organisms, carotenoids are structural components of the photosynthetic apparatus. Moreover, they possess specialized storage structures that allow them to accumulate secondary carotenoids in large intracellular pools. The high carotenoid yield of microalgae with respect to *W. schleiferi* is in part due to this role. However, even if microalgae normally produce higher yields, they need light and CO_2_ supply, which is not the case for non‐phototropic bacteria.

Additionally, in the interest of producing pure zeaxanthin, *W. schleiferi A* and *B* would be a more adequate choice, since microalgae normally produce lutein simultaneously [13]. Both strains produced only zeaxanthin as the presence of lutein was not detected. This facilitates purification given that separating lutein and zeaxanthin is challenging due to their similar chemical structures. Therefore, these *W. schleiferi* strains may be more useful for processes oriented towards high‐value end‐uses of zeaxanthin like macular degeneration and photo‐induced ocular fatigue treatment [3]. Moreover, these pharmaceutical applications are primarily concerned by the *all‐trans* isomer, as the structural form found in the macula region of the human retina consists primarily of the *all‐trans* isomer with low concentrations of the *cis* isomers [40]. As demonstrated by the present work, the zeaxanthin produced by *W. schleiferi* is mostly found in the *all‐trans* configuration with a small proportion of the *cis* isomer.

Genetically modified microorganisms for optimal zeaxanthin production have also been reported with yields of up to 300 mg L^−1^ [41, 42]. However, natural microorganisms possess several advantages for commercial production like no risk of environment contamination with modified bacteria and fewer commercial regulations.

To conclude, two novel bacterial strains belonging to *W. schleiferi* were isolated for the first time from the Mediterranean Sea. Nonetheless, they presented certain differences with respect to the *W. schleiferi* type strain Z215, like β‐glucosidase activity and the assimilation of additional saccharides, which grants them a greater adaptability in nutrient uptake.

The main carotenoid synthesized by strains A and B was identified to be zeaxanthin. Its production followed a similar trend to biomass growth suggesting it may be a primary metabolite. The specific production was 0.7 and 0.9 mg zeaxanthin per g of dry biomass for strains A and B, respectively.

Our findings can attract more interest in the genus *Winogradskyella* as sustainable candidates for biotechnological carotenoid production. Particularly, it was shown that *W. schleiferi* strains A and B could be applied in the production of zeaxanthin. In the future, further experiments will be necessary to focus on optimizing the biosynthesis of this valuable pigment. Specifically, by testing different nutrient sources and investigating abiotic stress factors.

## Author Contributions

**Aldo Betancourt Sanchez:** conceptualization, investigation, formal analysis, methodology, validation, visualization, writing – original draft, writing – review and editing. **Alexander Huynh:** investigation, validation, visualization, writing – review and editing. **Laurence Blanchard:** investigation, funding acquisition, visualization, writing – review and editing. **Arjan de Groot:** writing – review and editing, visualization, funding acquisition, investigation. **Christophe Klopp:** formal analysis, writing – review and editing. **Karine Loubière:** conceptualization, funding acquisition, methodology, supervision, visualization, validation, writing – review and editing. **Caroline Andriantsiferana:** conceptualization, methodology, validation, funding acquisition, supervision, visualization, writing – review and editing. **Barbora Lajoie:** conceptualization, funding acquisition, methodology, project administration, supervision, resources, visualization, validation, writing – review and editing.

## Conflicts of Interest

The authors declare no conflicts of interest.

## Supporting information


Supporting File

## Data Availability

The data that support the findings of this study are available in the supporting information of this article.
