# Supplementary material for: Zeaxanthin‐Producing Winogradskyella schleiferi Strains
Source: J Basic Microbiol. 2026 Jul 17;66(7):e70187. doi: 10.1002/jobm.70187 (PMC13377669; doi:10.1002/jobm.70187)
Supplement: Supplementary file 1 — Supporting File [file JOBM-66-e70187-s001.docx]

**Supporting Information**

**Zeaxanthin-producing *Winogradskyella schleiferi* strains**

Authors:

- Aldo Betancourt Sanchez. **Affiliation:** Université de Toulouse, Toulouse INP, CNRS, Laboratoire de Génie Chimique (LGC), Toulouse, France. **E-mail address:** [aldo.betancourtsanchez@toulouse-inp.fr](mailto:aldo.betancourtsanchez@toulouse-inp.fr) **ORCID ID:** 0009-0007-5729-2541
- Alexander Huynh. **Affiliation :** Université de Toulouse, Toulouse INP, CNRS, Laboratoire de Génie Chimique (LGC), Toulouse, France. **E-mail address:** [alexanderky.huynh@toulouse-inp.fr](mailto:alexanderky.huynh@toulouse-inp.fr) **ORCID ID:** 0009-0004-2272-6707
- Laurence Blanchard. **Affiliation:** Aix Marseille Univ, CEA, CNRS, BIAM, Molecular and Environmental Microbiology (MEM) Team, Saint Paul-Lez-Durance, F-13115, France. **E-mail address:** [laurence.blanchard@cea.fr](mailto:laurence.blanchard@cea.fr) **ORCID ID:** [0000-0003-1954-6137](https://orcid.org/0000-0003-1954-6137)
- Arjan de Groot. **Affiliation:** Aix Marseille Univ, CEA, CNRS, BIAM, Molecular and Environmental Microbiology (MEM) Team, Saint Paul-Lez-Durance, F-13115, France. **E-mail address:** [nicolaas.degroot@cea.fr](mailto:nicolaas.degroot@cea.fr) **ORCID ID:** [0000-0003-2202-5279](https://orcid.org/0000-0003-2202-5279)
- Christophe Klopp. **Affiliation:** INRAE, Genotoul Bioinformatics Platform, Applied Mathematics and Informatics of Toulouse, Sigenae, MIAT, UR875, Castanet Tolosan, France. **E-mail address:** [christophe.klopp@inrae.fr](mailto:christophe.klopp@inrae.fr) **ORCID ID:** 0000-0001-7126-5477
- Karine Loubière. **Affiliation:** Université de Toulouse, Toulouse INP, CNRS, Laboratoire de Génie Chimique (LGC), Toulouse, France. **E-mail address:** [karine.loubiere@cnrs.fr](mailto:karine.loubiere@cnrs.fr) **ORCID ID:** 00000-0001-6245-2844
- Caroline Andriantsiferana. **Affiliation:** Université de Toulouse, Toulouse INP, CNRS, Laboratoire de Génie Chimique (LGC), Toulouse, France. **E-mail address:** [caroline.andriantsiferana@iut-tlse3.fr](mailto:caroline.andriantsiferana@iut-tlse3.fr) **ORCID ID:** 0000-0002-6841-6403
- Barbora Lajoie. **Affiliation**Université de Toulouse, Toulouse INP, CNRS, Laboratoire de Génie Chimique (LGC), Toulouse, France. **E-mail address:** [barbora.lajoie@utoulouse.fr](mailto:barbora.lajoie@utoulouse.fr) **ORCID ID:** [0000-0003-0025-9496](https://orcid.org/0000-0003-0025-9496) *Corresponding author

**
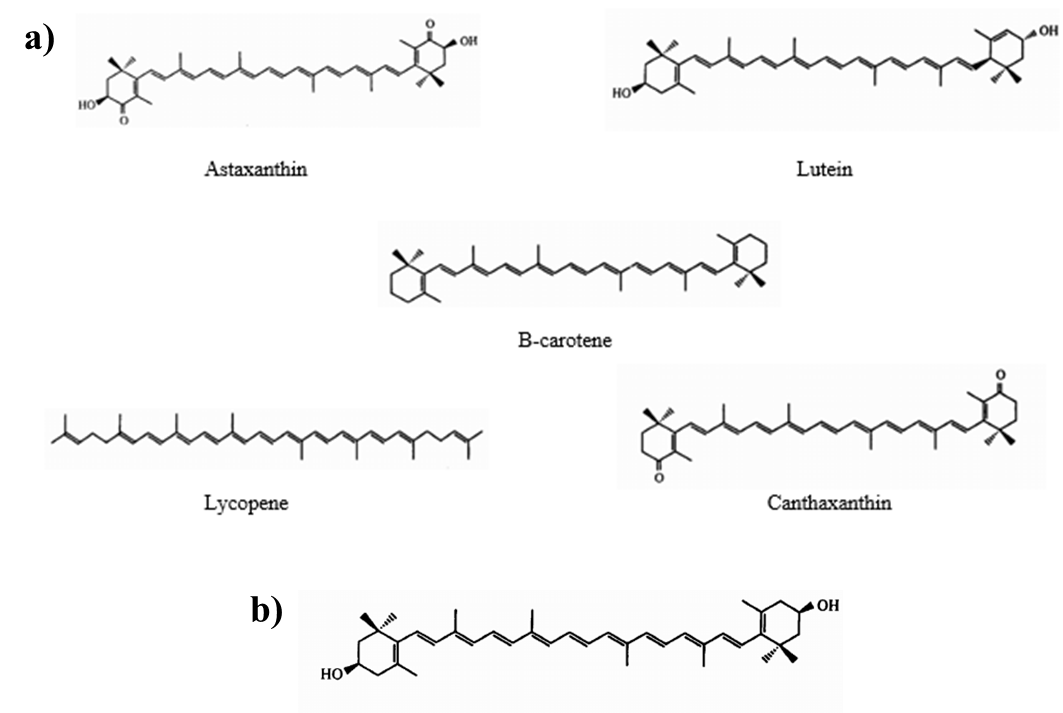
**

**Figure S1** Chemical structure of (a) the most industrially produced carotenoids and (b) zeaxanthin. Figure S1a presents the chemical structure of the most industrially produced carotenoids. Except β-carotene and lycopene, they belong to the xanthophyll family just like zeaxanthin itself (Figure S1b).

**Table S1**  Commercial marine broth (Difco^®^) composition.

| **Compound** | **Concentration (g L^-1^)** |
| --- | --- |
| NaCl | 19.00 |
| MgCl_2_ | 5.90 |
| Peptone | 5.00 |
| Yeast extract | 1.00 |
| Na_2_SO_4_ | 3.24 |
| CaCl_2_ | 1.80 |
| KCl | 0.55 |
| NaHCO_3_ | 0.16 |
| Iron citrate (III) | 0.10 |
| KBr | 0.080 |
| SrCl_2_ | 0.034 |
| H_3_BO_3_ | 0.022 |
| NaSiO_2_ | 0.004 |
| NaF | 0.0024 |
| NH_4_NO_3_ | 0.0016 |
| Na_2_HPO_4_ | 0.0080 |


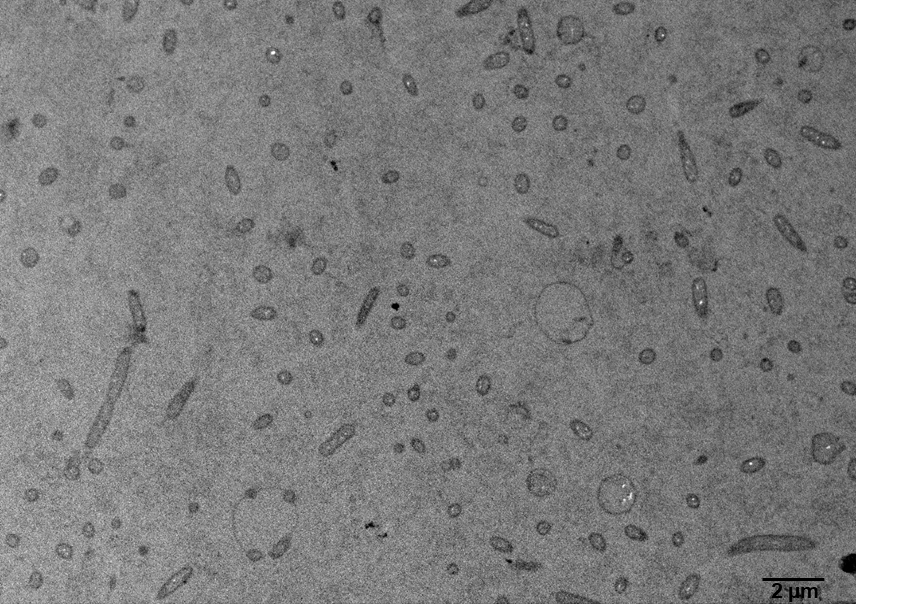


**Figure S2** Scanning electron microscopy of *W. schleiferi A*.

Figure S2 presents an electron microscopic picture of a culture of *W. schleiferi A*. Rod shapes are mainly observed with occasional coccus shapes appearing. The same pattern was observed for strain B.


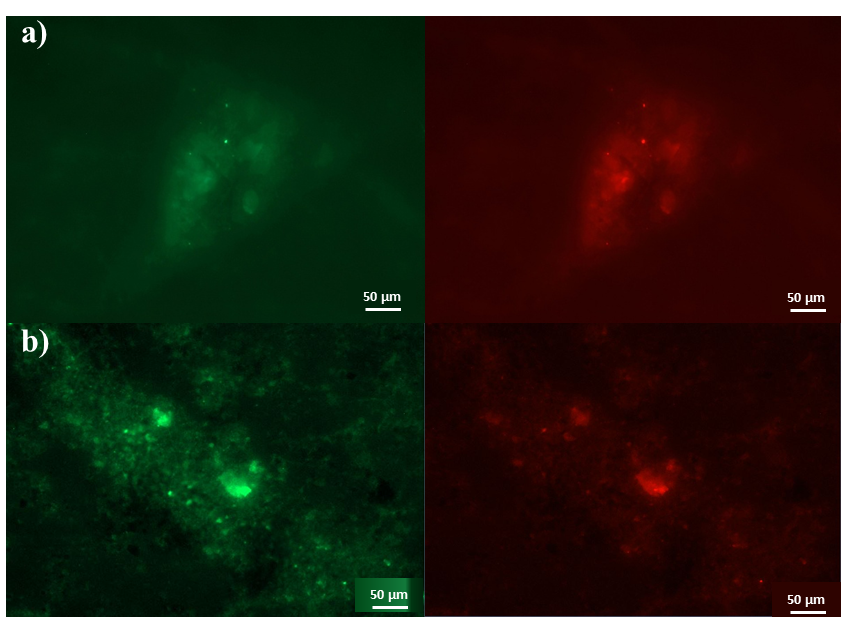


**Figure S3**  Epifluorescence microscopy pictures for a) strain A and b) B. Fluorescence emitted at 550 nm for green fluorescence and 630 nm for red fluorescence.

Figure S3 shows that both strains can be observed by epifluorescence microscopy attributable to the presence of carotenoids. When excited at 450 nm, both strains emitted green and red fluorescence detectable at 550 nm and 630 nm respectively.


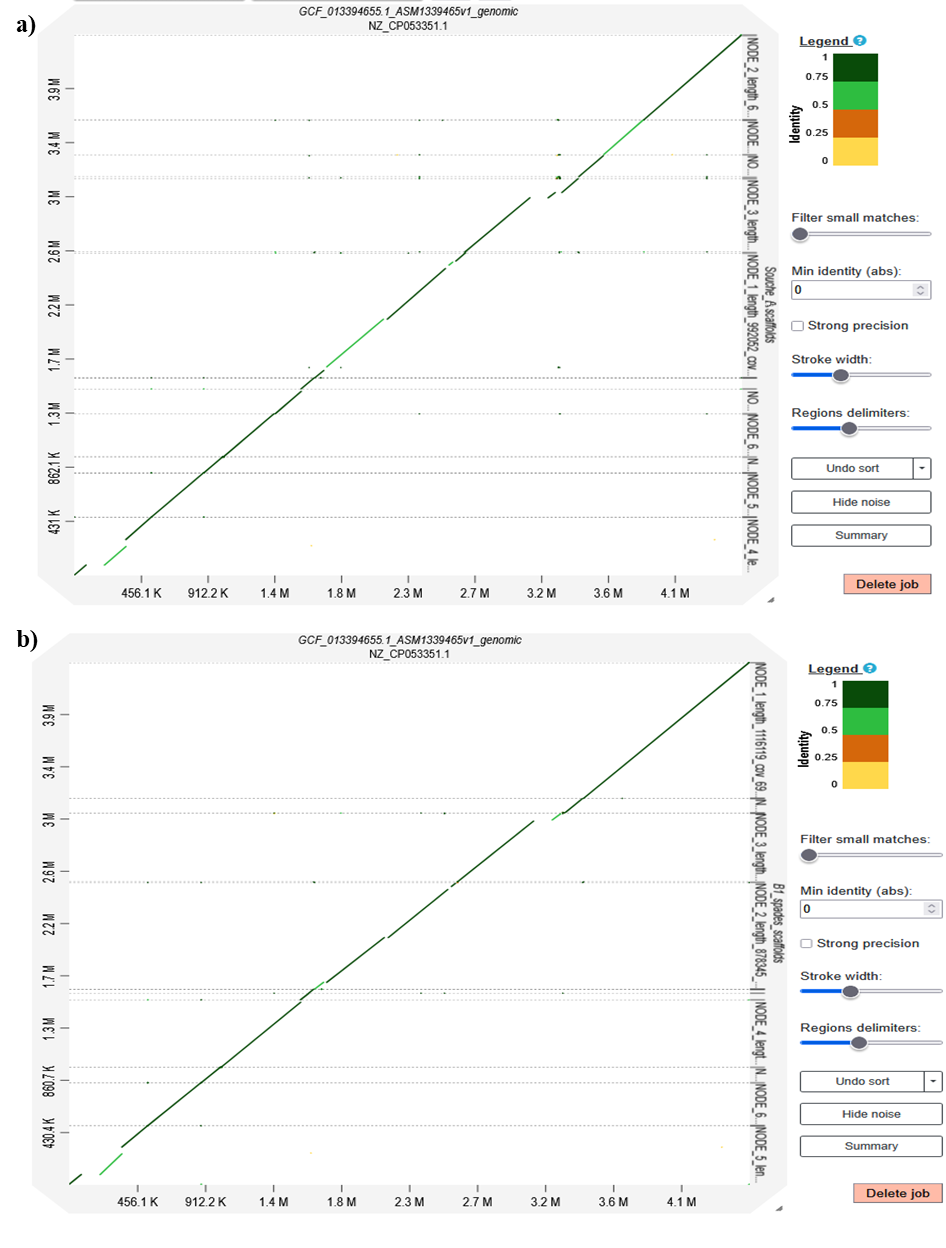


**Figure S4** a) Scaffold alignment comparison between *W. schleiferi* Z215 (GCF_013394655, horizontal axis) and strain A (vertical axis) and b) strain B (vertical axis) made with the D-GENIES software [19].

Figure S4 the resulting contigs and scaffold alignment analysis, which showed the correspondence between *W. schleiferi* Z215 and a) *Winogradksyella* strain A and b) strain B.

**Table S2** presents the comparison of the capacity of strains A and B to produce acid metabolites according to different carbon sources with respect to *W. exilis* and *W. marina*. The clearest difference was their inability to use glucose for this purpose. *W. schleiferi* Z215, *W. forsetii* and *W. ludwigii* were not included in the comparison as this information was not determined in the literature.

**Table S2** Comparison of acid production from different carbon sources by strains A and B with other *Winogradskyella* species, i.e. *W. exilis* [27], *W. marina* [28]. *n.d = non determined.

|  | Strain A | Strain B | *W. exilis 022-2-26* | *W. marina F6397* |
| --- | --- | --- | --- | --- |
| D-Glucose | - | - | + | + |
| D-mannose | + | + | + | + |
| D-mannitol | - | - | + | n.d. |
| L-arabinose | - | - | - | n.d. |
| Citrate | + | + | - | + |


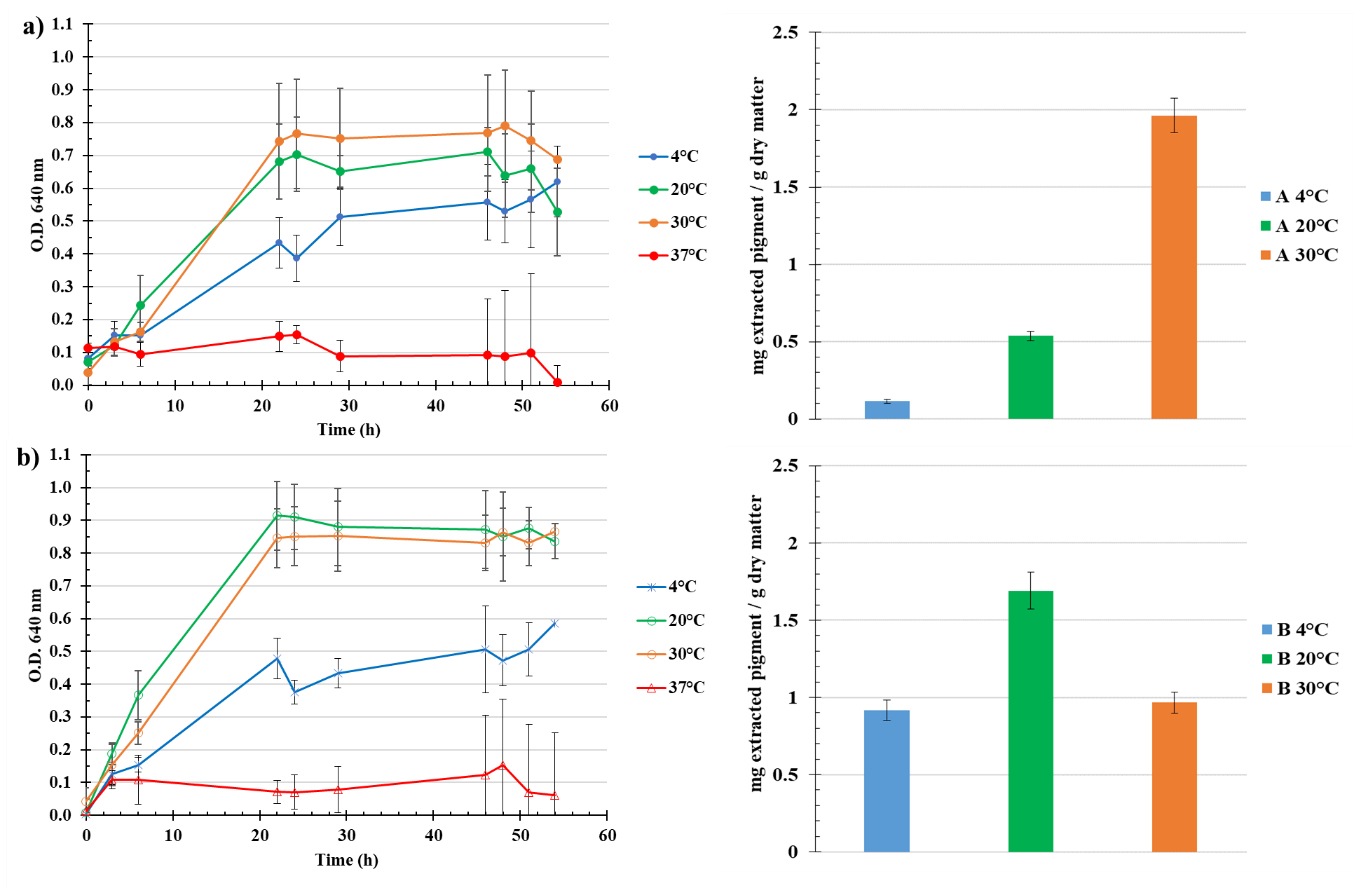


**Figure S5**  Growth of a) strain A and b) strain B measured by the evolution of optical density (OD) at 640 nm over time and at 4 different temperatures.

Figure S5 presents the results illustrating the influence of temperature on the growth and pigment production for strain A (a) and strain B (b). They both grew better at 20°C and 30°C with no significant difference between these two temperatures. However, the analysis of pigment specific production shows that strain A prefers 30° C (1.95 ± 0.15 mg g^-1^) and strain B 20°C (1.7 ± 0.15 mg g^-1^). No pigment production was observed at 37 °C, whatever the strain.


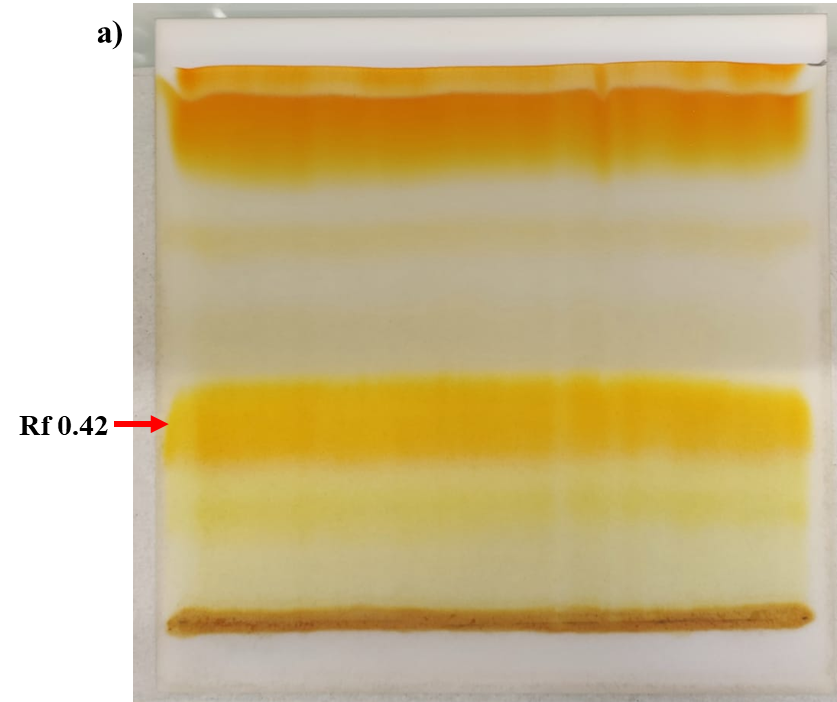


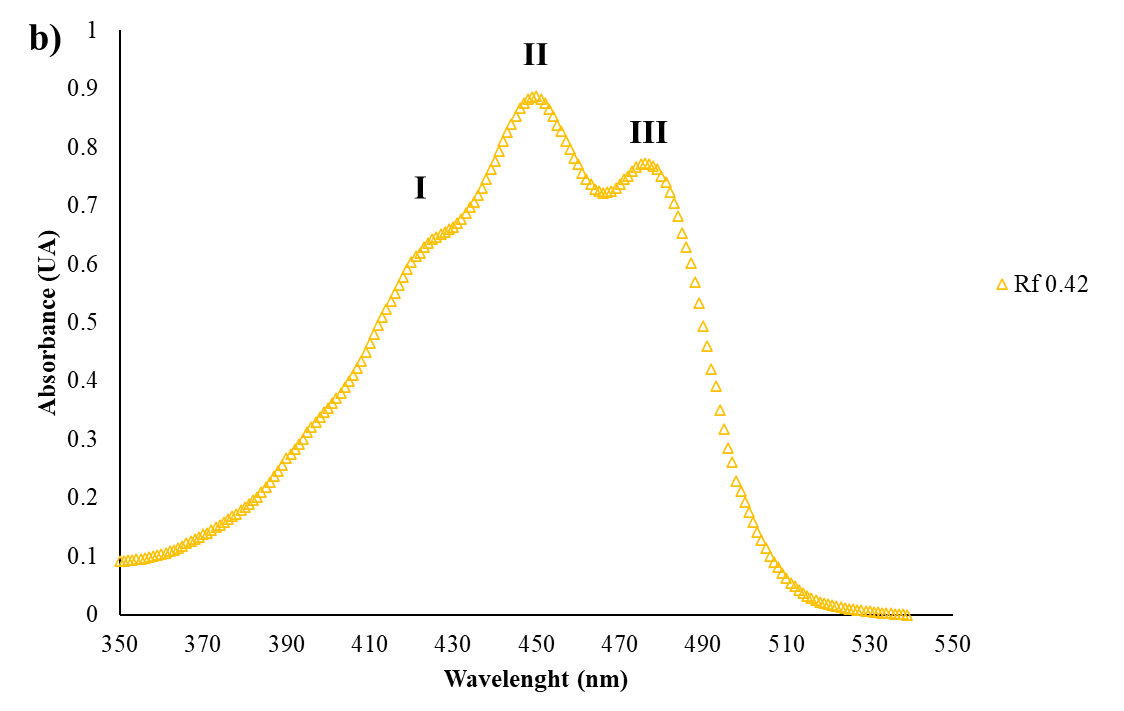


**Figure S6** a) Thin-layer chromatography silica plate after total carotenoid separation and b) absorbance spectrophotometry profile of the band with a Fr of 0.42, observed for both strains.

The large yellow band in the middle of the plate (Fr 0.42) observed in Figure S6a was recovered and separated from the silica layer, and then dissolved in ethanol to be analyzed by spectrometry. The spectra profile obtained (Figure S6b) was similar to that of zeaxanthin according to the bibliography, even sharing a similar %III/II ratio [25]. The sample was then prepared accordingly to analyze it by LC-MS.


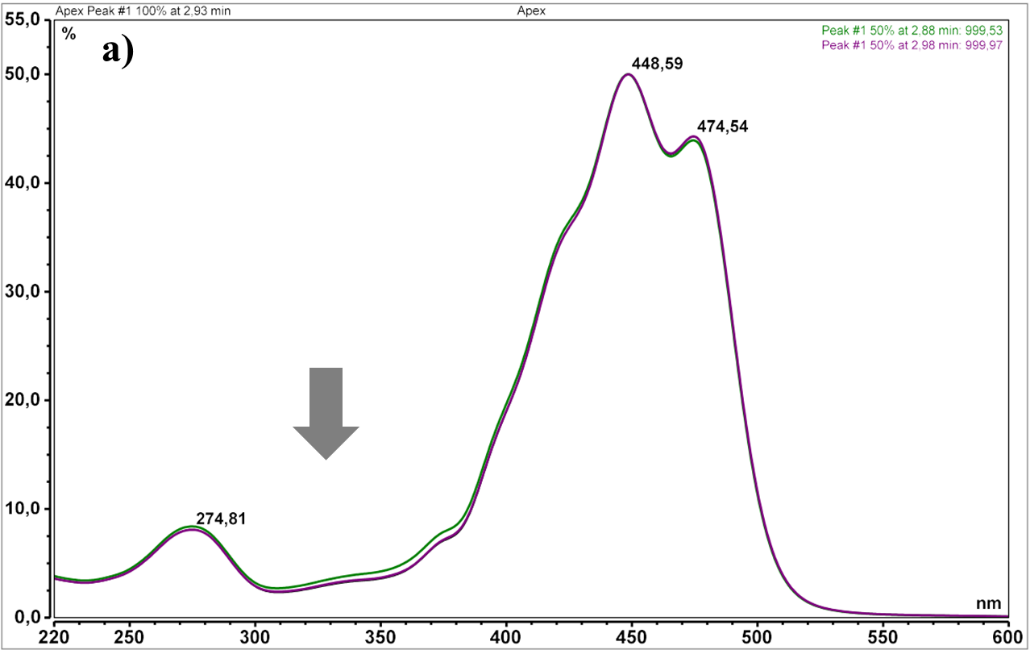

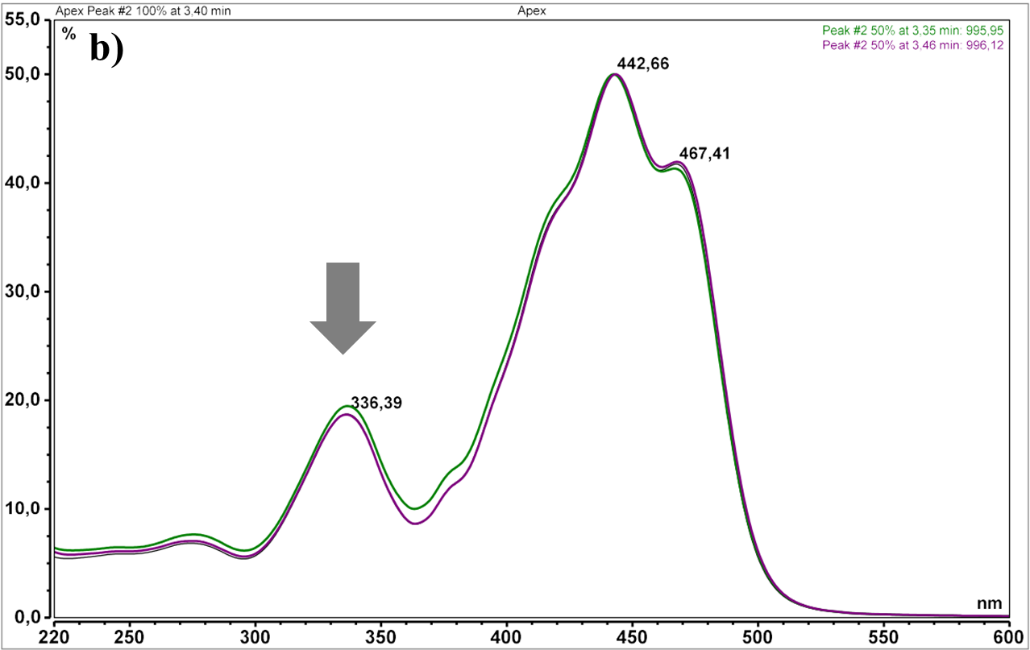


**Figure S7**  Absorption spectra profiles for a) peak 1 and b) peak 2 identified in the isolated pigment obtained (Fr 0.42).

Figure S7 illustrates the absorbance spectra profiles measured for the main peaks, of the Fr 0.42 pigment, detected by HPLC which correspond to the same profile as the isomers *all-trans*-zeaxanthin and *cis-*zeaxanthin.


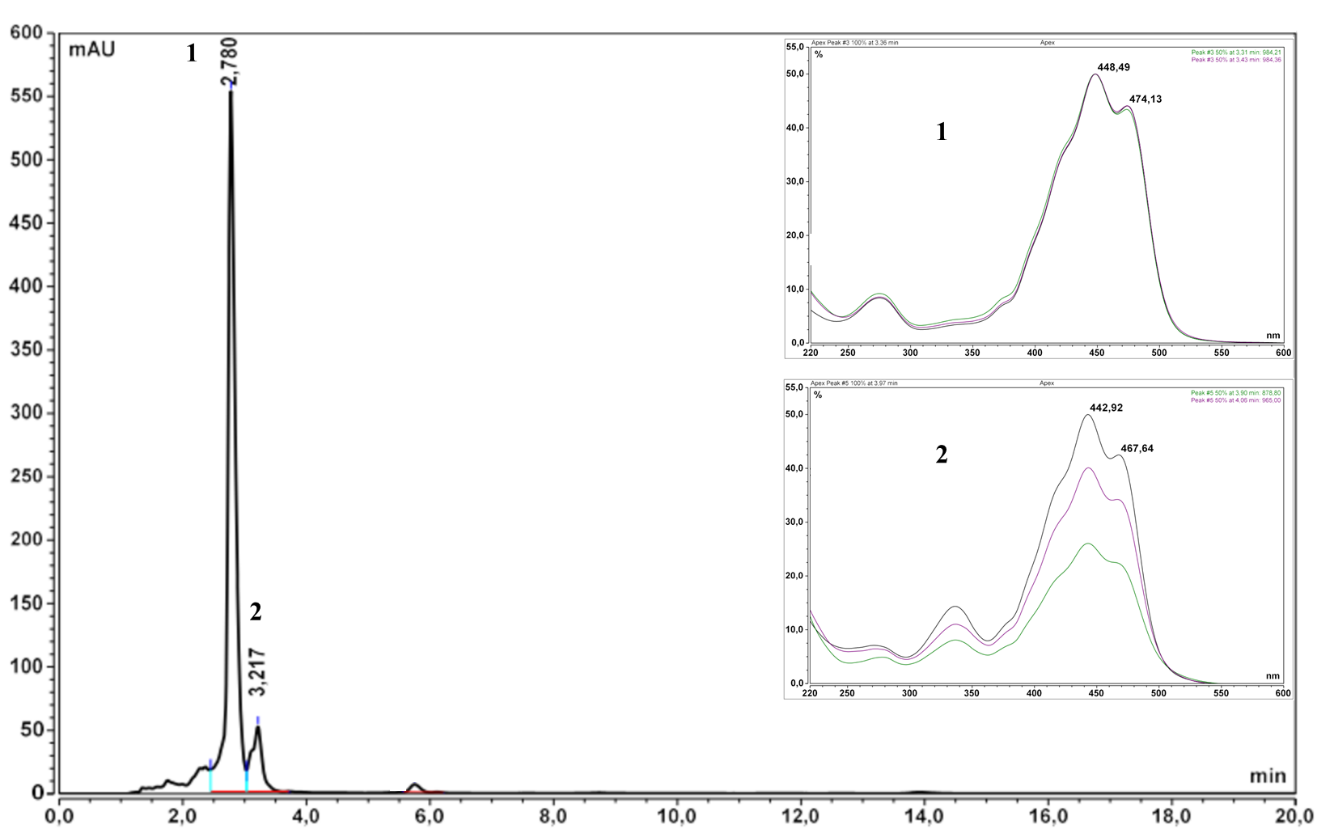


**Figure S8**  Example of an HPLC chromatogram and absorption spectra profiles of the total carotenoid extract produced by strain A.

In Figure S8, the main carotenoids detected in the total carotenoid extract of strain A (Rt 2.8, 3.2 min) are shown. The same main carotenoids were detected in Strain B with similar absorption spectra profiles (Figure 3).
